# The Extract of *Leonurus sibiricus* Transgenic Roots with AtPAP1 Transcriptional Factor Induces Apoptosis via DNA Damage and Down Regulation of Selected Epigenetic Factors in Human Cancer Cells

**DOI:** 10.1007/s11064-018-2551-6

**Published:** 2018-05-21

**Authors:** Przemysław Sitarek, Tomasz Kowalczyk, Simona Santangelo, Adam J. Białas, Monika Toma, Joanna Wieczfinska, Tomasz Śliwiński, Ewa Skała

**Affiliations:** 1 Department of Biology and Pharmaceutical Botany, Medical University of Lodz, ul. Muszyńskiego 1, 90-151 Lodz, Poland; 2 Department of Genetics and Plant Molecular Biology and Biotechnology, The University of Lodz, Banacha 12/13, Lodz, Poland; 3 Geriatrics, Department of Respiratory Pathophysiology, Campus Bio-Medico University and Teaching Hospital, Rome, Italy; 4 Department of Pneumology and Allergy, 1st Chair of Internal Medicine, Medical University of Lodz, Lodz, Poland; 5 Laboratory of Molecular Genetics, University of Lodz, Lodz, Poland; 6 Department of Immunopathology, Chair of Allergology, Immunology and Dermatology, Faculty of Biomedical Sciences and Postgraduate Training, Medical University of Lodz, Lodz, Poland

**Keywords:** TR and AtPAP1 roots of *Leonurus sibiricus*, *UHRF1* and *DNMT1* gene expression, DNA damage, Phosphorylation of H2A.X, Cleaved PARP1

## Abstract

The aim of this study was to determine the anticancer potential of *Leonurus sibiricus* extract derived from in vitro transgenic roots transformed by *Agrobacetrium rhizogenes* with AtPAP1 transcriptional factor, and that of transformed roots without construct, on grade IV human glioma cells and the U87MG cell line, and attempt to characterize the mechanism involved in this process. The anticancer effect induced by the tested extracts was associated with DNA damage, PARP cleavage/increased H2A.X histone levels and *UHRF-1*/*DNMT1* down-regulation of mRNA levels. Additionally, we demonstrated differences in the content of compounds in the tested extracts by HPLC analysis with ATPAP1 construct and without. Both the tested extracts showed anticancer properties and the better results were observed for AtPAP1 with transcriptional factor root extract; this effect could be ascribed to the presence of higher condensed phenolic acids such as neochlorogenic acid, chlorogenic acids, ferulic acid, caffeic acid and *p*-coumaric acid. Further studies with AtPAP1 (with the transcriptional factor from *Arabidopisi thaliana*) root extract which showed better activities in combination with anticancer drugs are needed.

## Introduction

Traditional medicinal plants have been used in treating cancer for several millennia in several parts of the globe and herbal medicines are currently being used for treating a variety of ailments worldwide, either alone or in combination with conventional therapeutics [1, 2]. Plant-based bioactive compounds are known to exert anti-cancer activities in various ways: altering the carcinogen metabolism, inducing DNA damage, activating the immune system, inhibiting cell cycle progression and inducing apoptosis. They are also known to possess chemotherapeutic and chemopreventive activities against cancer cells [3, 4]. One such plant is *Leonurus sibiricus* L, of the family Lamiaceae, which has been used in traditional medicine for hundreds of years. The active compounds in *L. sibirius*, such as phenolic acids and diterpenes possess various biological activities [5–9]. Our previous studies showed that transformed root extract of *Leonurus sibiricus* induces the extrinsic and intrinsic apoptosis pathways in glioma cells by altering the expression of antiapoptotic and proapoptotic genes [8]. Additionally, in this study we used an extract with transcriptional factor from *Arabidopsis thaliana* (AtPAP1) insert by transformation by *Agrobacterium rhizogenes* into *L. sibirius* roots which enhances the production of phenolic acids and may improve its biological properties [10]. The aim of this project was to better understand the mechanism of the anticancer effects on grade IV glioma cells and U87MG cells after treatment of *L. sibiricus* transformed root extract (TR) and transgenic root extract with transcriptional factor AtPAP1; these effects may be facilitated by increased DNA damage, PARP cleavage, H2A.X histone and *UHRF-1*/*DNMT1* regulation. PARP helps repair DNA damage and restores its activity in three ways: catalysing poly (ADP-ribose) synthesis, modifying nuclear proteins and binding to DNA strand breakage [11]. γ-H2A.X is the phosphorylated form of histone H2A.X, which appears at the site of DNA damage, particularly double SBs, and is a sensitive indicator of damage [12]. *UHRF1* is a nuclear protein which plays an important role in the development of cancer by epigenetic regulation. *DNMT1*, an enzyme that interacts with UHRF1, is also known to accumulate at DNA damage locations [12].

## Materials and Methods

### Material and Reagents

The U87MG cell line (89081402) was purchased from Sigma. EMEM (EBSS) medium was purchased from LONZA. TaqMan® Real-Time PCR Master Mix and *UHRF1, DNMT1, 18S RNA* genes were purchased from Life Technologies. Apoptosis, DNA Damage and Cell Proliferation Kit was purchased from BD Pharmingen (562253).

### Plant Material Obtained from *L. sibiricus* Transformed Roots (TR) and Transgenic Roots with Transcriptional Factor (AtPAP1)

The TR and AtPAP1 root cultures were established as described previously [7, 10], as was the PCR (polymerase chain reaction) protocol used to confirm TR root transformation with the *rol*B and *rol*C genes in T-DNA [13], and the confirmation of the AtPAP1 root transformation itself [10]. Briefly, about 10 g d.w. of lyophilized and powdered TR and AtPAP1 root was used. The yields (w/w) were 52.5% initial d.w. for the TR extract and 50.25% for the AtPAP1 extract [10].

### Analysis of Compounds by HPLC and LC–MS/MS Methods

Chemical analysis of TR and AtPAP1 root extracts with all conditions were performed as described earlier by Sitarek et al. [10]. Phenolic compounds were identified by LC-MS/MS and their contents were determined by HPLC according to Sitarek et al. [7].

### In Vitro Cell Cultures

Grade IV glioma cells derived from surgical specimens were maintained as described previously [7]. The U87MG cell line (89081402, Sigma) was cultured in EMEM (EBSS) medium (LONZA) supplemented with 10% Fetal Bovine Serum (FBS), as instructed by the manufacturer.

### RNA Isolation, cDNA Synthesis and Real-Time PCR

Total RNA isolation kit (A&A Biotechnology) was used for RNA isolation and purification, TranScriba Kit (A&A Biotechnology) was used to transcribe the RNA into cDNA. The TaqMan® Real-Time PCR Master Mix (Life Technologies) and Agilent Technologies Stratagene Mx300SP working on MxPro software were then used to perform qRT-PCR. Two genes (*UHRF1, DNMT1*), with *18S RNA* (Life Technologies) acting as a reference gene, were analysed using TaqMan probes (Life Technologies). The procedure was as follows: 95 °C for 10 min, 30 cycles of 95 °C for 15 s and 60 °C for 60 s.

### Analysis of Phosphorylated H2A.X and Cleaved PARP Levels

Grade IV glioma cells and U87MG cells were plated in a 6-well plate at a density of 2 × 10^5^ viable cells. The following day, TR and AtPAP1 root extracts were added at a concentration corresponding to 50% viability. After 24-h incubation, the cells were collected and phosphorylated H2A.X and cleaved PARP-positive cells were detected using Apoptosis, DNA Damage and Cell Proliferation Kit (BD Pharmingen, 562253) according to the protocol attached by the manufacturer. The cells were analyzed with a FACS Canto II cytometer (Becton Dickinson, USA).

Additionally, the level of phosphorylated histone γ-H2A.X was performed using an H2A.X Phosphorylation Assay Kit (Millipore, Billerica, MA, USA) according to the protocol. Chemiluminescence detection was performed using attached HRP-substrates using a GloMax-Multi device (Promega).

### Comet Assay Measurement of DSBs

The cells were treated with 0.3, 1 and 1.5 mg/ml of TR and AtPAP1 root extracts for up to 24 h before washing twice with 1 ml PBS and collecting into 1 ml PBS and analysed by a neutral version of the comet assay to detect DSBs, as described before with modifications according to Czyż et al. [14]. Briefly, cells were suspended in 0.75% LMP agarose and casted onto microscope slides precoated with 0.5% NMP agarose. The cells were then lysed for one hour at 4 °C in a buffer consisting of 2.5 mM NaOH, 100 mM EDTA, 1% Triton X-100, 10 mM Tris, pH 10. After lysis, the slides were placed in an electrophoresis unit, DNA was allowed to unwind for 20 min in an electrophoresis buffer consisting of 100 mM Tris and 300 mM sodium acetate at a pH adjusted to 9.0 by glacial acetic acid. Electrophoresis was conducted in this electrophoresis buffer at 4 °C for 60 min at an electric field strength of 0.41 V/cm (100 mA). The slides were then washed in water, drained and stained with 2 µg/ml of DAPI and examined with a microscope image analysis system: This entire system comprised an Eclipse fluorescence microscope (Nikon, Tokyo, Japan) attached to a COHU 4910 video camera (Cohu, San Diego, CA, USA) equipped with a UV-1 filter block, consisting of an excitation filter (359 nm) and a barrier filter (461 nm); this setup was connected to a Lucia-Comet v. 5.41 personal computer-based image analysis system (Laboratory Imaging, Praha, Czech Republic). Fifty images were randomly selected from each sample and the percentage of DNA in the tail of comets (% tail DNA) was measured. The mean value of the % tail DNA in a particular sample was taken as an index of DSBs in the sample.

### Statistical Analysis

All experiments were performed in triplicate. The results are expressed as mean ± SD. Shapiro–Wilk test was used to verification the data normality. The Kruskal–Wallis test with multiple comparisons of average ranks and the one-way analysis of variance (ANOVA) and the subsequent Tukey post hoc test were used to determine differences between samples (*p* < 0.05).

## Results

### Establishment and Genetic Characterization of *L. sibiricus* Transgenic Root with Arabidopsis AtPAP1 Construct and TR Roots

The AtPAP1 with gene under the control of the pCAMBIA 1305.1 promoter was transferred into *L. sibiricus* by *A. rhizogenes* as described previously [10]. Confirmation of genetic transformation was performed with *hptII* specific primers for the AtPAP1 root extract and with the *rol*B and *rol*C genes to T-DNA for the TR root extract as described previously by Sitarek et al. [10].

### HPLC Analysis of Phenolic Acids in AtPAP1 and TR Root Extracts

The quantitative determination of phenolic acids in TR and AtPAP1 extracts was performed as described previously [10]. Briefly, HPLC analysis indicated that all phenolic acids (neochlorogenic acid, chlorogenic acid, caffeic acid, *p*-coumaric acid and ferulic acid) were present in greater amounts in the AtPAP1 root extract than the TR roots without construct. The first dominant phenolic acid in both extracts was chlorogenic acid. The chlorogenic acid content in the AtPAP1 root extract was 19392 µg of dry weight, i.e. 4.7 times higher than in the TR root extract (4104 µg/g of dry weight) (*p* < 0.05). The second phenolic acid, i.e. caffeic acid, constituted 11380 µg/g of dry weight in the AtPAP1 root extract, i.e. 2.7-times higher than that in the TR root extract (4176 µg/g of dry weight). Results are shown in Table 1.

**Table 1:** The contents of phenolic acids in *Leonurus sibiricus* TR and AtPAP1 transformed root extracts

No.	Phenolic compounds	TR extract µg/g DW	AtPAP1 extract µg/g DW
1	Neochlorogenic acid	8 ± 0.4^a^	18 ± 6.0^b^
2	Chlorogenic acid	4104 ± 8.7^a^	19392 ± 110.1^b^
3	Caffeic acid	4176 ± 9.0^a^	11380 ± 136.6^b^
4	*p*-Coumaric acid	30 ± 0.1^a^	52 ± 1.1^b^
5	Ferulic acid	660 ± 27.1^a^	1172 ± 36.3^b^

The phenolic acids were determined in 80% aqueous methanol extracts from TR (used as the control) and transgenic roots with AtPAP 1 transcriptional factor (AtPAP1). Different superscript letter within the rows indicates significant differences in the mean values at *p* < 0.05

### Dose-Dependent Inhibition of Cell Viability After Treatment with *L. sibiricus* TR and AtPAP1 Root Extracts

**Fig. 1:**
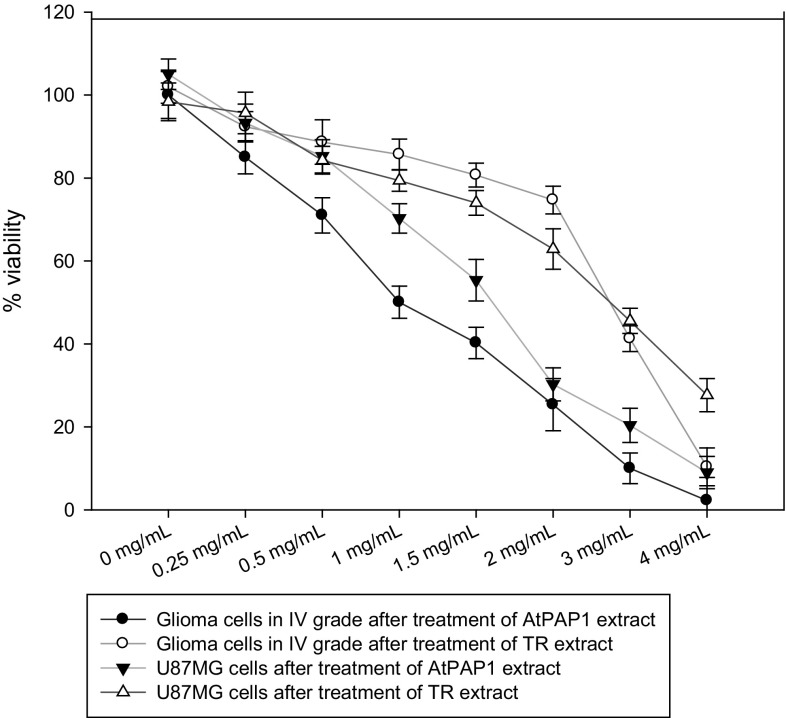
Glioma cells in IV grade and U87MG cells were exposed to varying concentrations (0–4 mg/ml) of *L. sibiricus* TR and AtPAP1 root extracts. Viability cells was assessed after 24 h by MTT test. The values represent mean ± SD of three independent experiments

As shown in Fig. 1, TR and AtPAP1 root extracts of *L. sibiricus* reduced the viability of treated grade IV glioma cells and U87MG cells in a dose-dependent manner. Based on the cell viability measured after 24 h, the approximate 50% inhibitory concentrations of TR and AtPAP1 *L. sibiricus* root extracts for grade IV glioma cells were approximately 2.5 and 1 mg/ml, respectively. In turn, TR and AtPAP1 root extract concentrations were approximately 2.5 and 1.5 mg/ml for U87MG cells, respectively. The tested cancer cell lines were the most sensitive after treatment with AtPAP1 *L. sibiricus* root extracts.

### DNA Damage Measured by Comet Assay

**Fig. 2:**
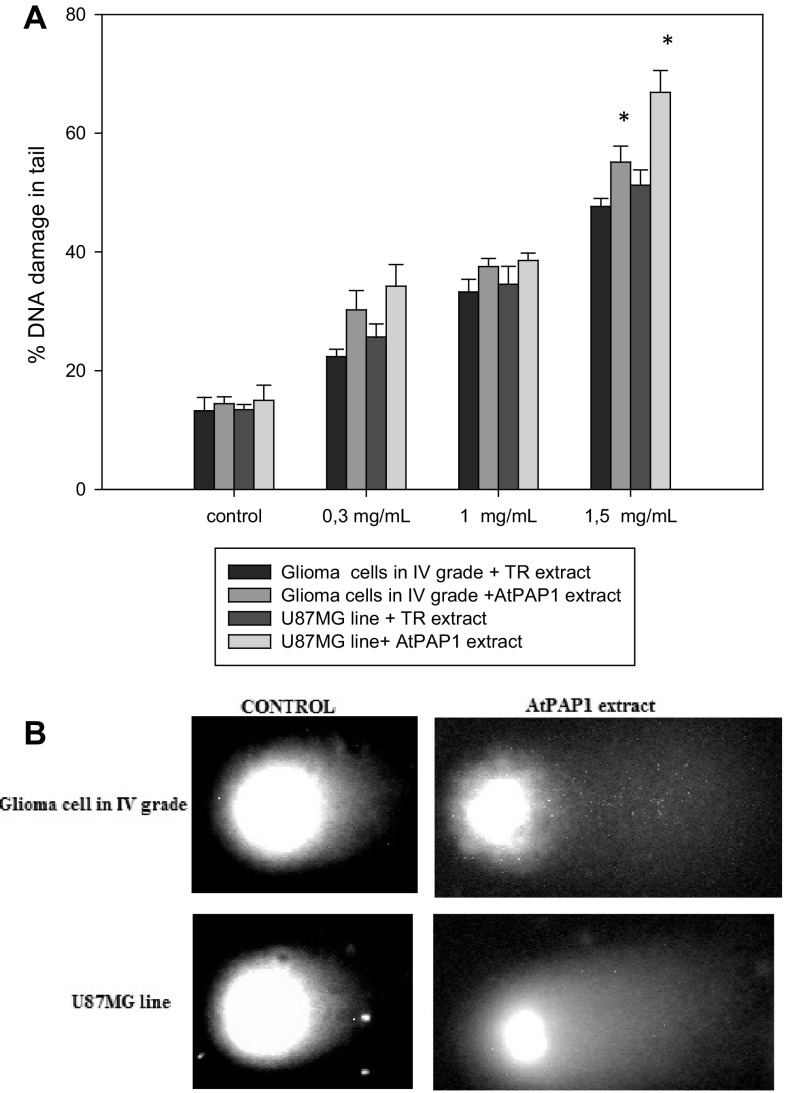
**a** Analysis of DNA damage as measured by comet assay in glioma cells in IV grade and U87MG cells treated with *L. sibirius* TR and AtPAP1 root extracts after 24 h and untreated cells (used as the control). **b** Representative images of comet assay. Each values represent mean ± SD of three separate experiments. *p < 0.05 AtPAP1 extract vs. TR extract for the same cell lines

Furthermore, we examined the DNA damage induced by TR and AtPAP1 root extract of *L. sibiricus* after 24 h of incubation. Induction of DNA strand breaks was dose dependent in both cell lines (Fig. 2) and the percentage of DNA strand breaks was approximately the various for each concentration used. Level of total DNA damage was about 65% for the higher concentration in U87MG cells after treatment of AtPAP1 root extract. U87MG cells were less sensitive to DNA damage compared with the glioma cells after treatment with AtPAP1 root extract.

### PARP1 and H2A.X Level Measured by Flow Cytometry

**Fig. 3:**
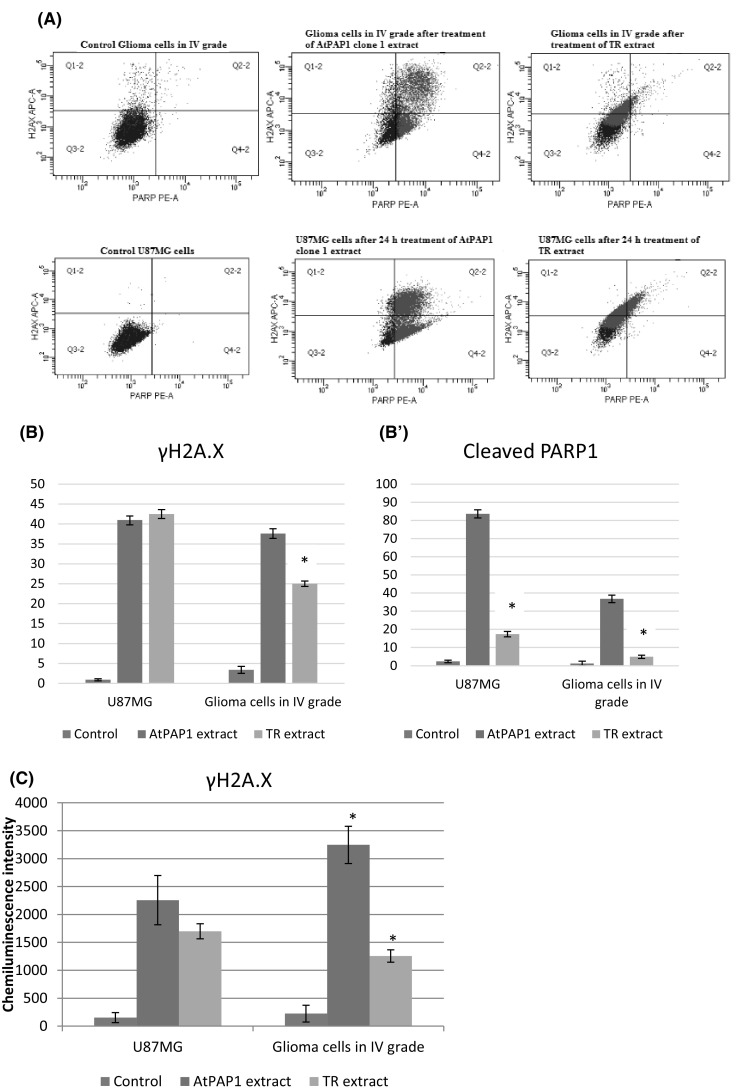
Representative histograms after flow cytometric analysis of phosphorylated H2AX and cleaved PARP1-positive cells (**a**). Diagrams presented percentage of γ-H2AX (**b**), cleaved PAPR1-positive cells (**b′**) and the level of γ-H2AX measured by Elisa test (**c**) after treatment TR and AtPAP1 of *L. sibiricus* root extracts in glioma cells in IV grade and U87MG cells. The values represent mean ± SD of three independent experiments. *p < 0.05 AtPAP1 extract vs. TR extract

In order to obtain data concerning the number of cells expressing the apoptosis marker, i.e. cleaved Poly ADP-Ribose Polymerase 1 (PARP1), flow cytometry analysis was performed on both U87MG cell line and grade IV glioma cells (Fig. 3a). After 24-h incubation with the analysed TR and AtPAP1 root extracts, the percentage of cleaved PARP1-positive cells increased significantly in both cell lines. Although both extracts elevated the content of apoptotic cells, AtPAP1 root extract was more efficient in this regard, raising the level of PARP1-positive cells that of the TR root extract (Fig. 3b). In the same experiment, the content of γH2A.X-expressing cells was accessed to reveal whether the analysed extracts induced DNA double-strand breaks and induced the action of DNA repairing machinery. In the U87MG cell line, the level of γH2A.X- positive cells did not differ between the treatment variants but in patient-derived grade IV glioma cells, AtPAP1 caused slightly higher elevation of the number of cells expressing the analysed marker (Fig. 3b′). Additionally, the level of γH2A.X was measured by Elisa test. The results indicated that TR and AtPAP1 root extracts of *L. sibiricus* increased the level of γH2A.X in both tested cell lines (Fig. 3c).

### Gene Expression

**Fig. 4:**
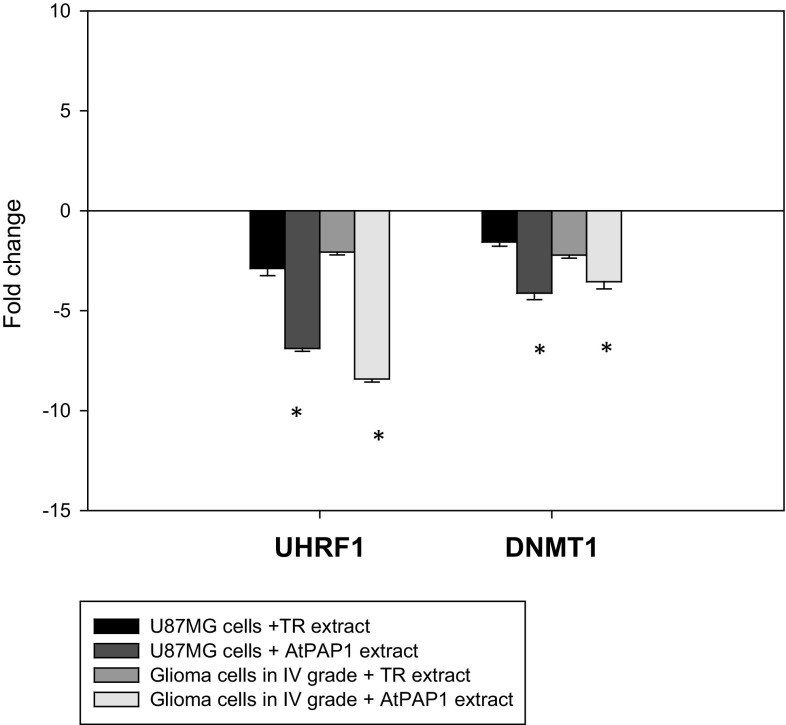
Expression profiles of UHRF1 and DNMT1 genes. qRT-PCR analysis of UHRF1 and DNMT1 in glioma cells in IV grade and U87MG cells cultured for 24 h in the presence of *L. sibiricus* TR and AtPAP1 root extracts. Each gene was normalized to the expression of a 18S RNA- reference gene. Data is presented as fold change in glioma cells in IV grade and U87MG cells vs. untreated cells, in which expression levels of the genes were set as 1. The mean values ± SD were calculated from three independent experiments. *p < 0.05 AtPAP1 extract *vs*. TR extract for the same cell lines

The expression of several apoptosis-related genes was determined by qRT-PCR analysis of grade IV glioma cells and U87MG cells exposed to TR and AtPAP1 root extracts of *L. sibiricus*. As depicted in Fig. 4, both plant extracts (with IC_50_ concentration for grade IV glioma cells and U87MG cells) showed down-regulation of *UHRF1* and *DNMT1* gene expression after treatment with TR and AtPAP1 root extracts on both tested lines. Better results were noticed for AtPAP1 root extract compared to TR extract in all tested genes (Fig. 4).

## Discussion

Medicine is constantly looking for a new mechanisms and strategies which may be the key to inducing the apoptosis in cancer cells [15]. Disturbances of this process in cancer cells have been studied in detail, and induction of apoptosis is one of the strategies for anticancer drug development [16, 17]. Plant extracts and their bioactive compounds are becoming useful resources for developing less toxic and more effective drugs to manage cancer progression [18]. In addition, biotechnology has also played a large role in the past few years, by allowing the genetical manipulation of plants to increase the production of effective compounds for low-cost treatment [19].

Our findings indicate that both tested extracts induced apoptosis in glioma cell lines in a concentration-dependent manner, but the AtPAP1 root extract demonstrated the better results. We assume that these differences can be caused by the different content of the compounds present in the extracts. The quantitative HPLC analysis revealed a higher amount of phenolic acids (chlorogenic acid, caffeic acid, ferulic acid, *p*-coumaric acid) in the AtPAP1 root extract than the TR root extract [10]. These properties can be attributed to the pCAMBIA1305.1-AtPAP1 vector which contains a transcriptional factor from *Arabidopsis thaliana* introduced by genetic engineering. This factor is known to increase the production of phenolic acids [20] which is consistent with our earlier research [10]. Our first test showed that both tested extracts may induce double-strand breaks in glioma cells, detectable by comet assay after 24-h treatment. The DNA-damaging effect might be induced by the phenolic acid content in the two sets of tested extracts. It has been suggested that phenolic compounds may intercalate with DNA, resulting in the mutagenic and pro-oxidant effects seen in the present study [21]. Our results are agreement with Koňariková et al which showed that black tea extract (BTE) rich in polyphenolic compounds induced also DNA strand breaks and oxidative damage to DNA in HT-29 and MCF-7 carcinoma cells [22]. Similarly, Aslanturk and Celik demonstrated that *Euphorbia platyphyllos* L extract (rich in polyphenolic compounds) induced significant DNA damage in MCF-7 breast cancer cells [23]. In turn, Burgos-Morón et al. revealed that phenolic acid (chlorogenic acid) caused increased DNA damage in A549 lung cancer cells [24]. In our studies one of the main compounds contained in the TR and AtPAP1 root extracts was chlorogenic acid, which can confirm our earlier hypothesis. This DNA damage response pathway may be the route by which apoptosis can be induced in cancer cells.

Additionally, flow cytometry analysis revealed that this effect can be related to PAPR1 cleavage and an increase of γ-H2A.X level. PARP1 is believed to maintain genomic integrity by repairing DNA repair, and its cleavage is an indicator of apoptosis [25]. Another clear indicator of apoptosis is PARP-1 activation, which also increases the rate of cellular disassembly [11]. Excessive activation of PARP-1 following extensive DNA damage is an indicator of NAD+ (a PARP-1 substrate) and ATP depletion; this depletion induces a dramatic reduction of cellular energetic pools, typically seen during apoptosis [11], resulting in cell dysfunction and cell death [26]. Our results are agreement with those of Krifa et al., which demonstrate that the polyphenolic extract of *L. guyonianum* enhances apoptotic effects in U373 cells via DNA damage and PARP cleavage [27]. However, Robles-Escajeda et al. showed that GB (green barley) extract induced apoptosis in the Nalm-6 cells by a caspase-3 activation →PARP-1 cleavage cascade [28]. This confirms our previous studies which revealed that the tested extract activates cascade of caspases [8]. Furthermore, Lin et al. showed that *Ginkgo biloba* polyphenolic extract caused a dose-dependent increase DNA double-strand breaks (DSBs) and activated the DNA damage signalling pathway with increased γH2A.X levels in L5178Y cells [29].

The next step in our study checked *UHRF1* and *DNMT1* expression after 24-h treatment with TR and AtPAP1 root extracts of *L. sibiricus*. Our findings reveal the down-regulation of the *UHRF1* and *DNMT1* genes. *UHRF1* is an ubiquitin-like protein which contains PHD (plant homeodomain) and RING finger domains. It plays a fundamental role in DNA epigenetic marker inheritance [30] and activates the pro-apoptotic pathway inhibiting the proliferation and transformation of cells, and preventing tumour vascularization [31]. Moreover, *UHRF1* interacts with *DNMT1* (DNA methyltransferase 1); this methylates the cytosine residues present on CpG islands of hemimethylated DNA [32–34]. The DNA of cells lacking *UHRF1* are known to be hypersensitive to damage from genotoxic agents, indicating that *UHRF1* helps maintain the integrity of the genome. Krifa et al. found that polyphenolic extract of *Limoniastrum guyonianum* induced apoptosis in a human cervical cancer HeLa cell line, probably via the activation of a p^16INK4A^ -dependent cell cycle checkpoint signalling pathway orchestrated by *UHRF1* and *DNMT1* down-regulation [35]. Similarly, Achour et al. report that epigallocatechin-3-gallate (polyphenolic compound) induces apoptosis in Jurkat cells by UHRF1 downregulation and p^16INK4A^ upregulation [36]. In turn, Alhosin et al. note that bilberry extract rich in polyphenols caused reactive oxygen species (ROS)- dependent but *p53*-/*p73*-independent UHRF1 downregulation in chronic lymphocytic leukaemia cells [37]. Therefore, it is possible that the high phenolic acid content in our tested TR and AtPAP1 root extracts may cause down-regulation of *UHRF1* and *DNMT1*, but further studies are needed.

## Conclusion

We demonstrate for the first time that TR and AtPAP1 root extracts of *Leonurus sibiricus* containing high amounts of phenolic acid may activate specific molecular signalling pathways and significantly interfere with the survival and proliferation of grade IV glioma cells and U87MG cancer cells by two routes: firstly, by inhibition of PARP activity, which increases the susceptibility of cells to DNA damaging agents, possibly by preventing DNA strand break rejoining; secondly, by decreasing *UHRF1* and *DNMT1* expression, thus influencing epigenetic regulation. Further investigations are needed with AtPAP1 root extract (with the transcriptional factor from *Arabidopsis thaliana*), which demonstrated better results in combination with anticancer drugs.

## References

[1] Sultana S, Asif HM, Nazar HM, Akhtar N, Rehman JU, Rehman RU (2014). Medicinal plants combating against cancer–a green anticancer approach. Asian Pac J Cancer Prev.

[2] Poojari RJ, Patil AG, Gota VSJ (2012). Development of botanical principles for clinical use in cancer: where are we lacking?. Postgrad Med.

[3] Skała E, Sitarek P, Toma M, Szemraj J, Radek M, Nieborowska-Skorska M, Skorski T, Wysokińska H, Śliwiński T (2016). Inhibition of human glioma cell proliferation by altered Bax/Bcl-2-p53 expression and apoptosis induction by Rhaponticum carthamoides extracts from transformed and normal roots. J Pharm Pharmacol.

[4] Rezaei PF, Fouladdel S, Hassani S, Yousefbeyk F, Ghaffari SM, Amin G, Azizi E (2012). Induction of apoptosis and cell cycle arrest by pericarp polyphenol-rich extract of Baneh in human colon carcinoma HT29 cells. Food Chem Toxicol.

[5] Lee MJ, Lee HS, Park SD, Moon HI, Park WH (2010). *Leonurus sibiricus* herb extract suppresses oxidative stress and ameliorates hypercholesterolemia in C57BL/6 mice and TNF-alpha induced expression of adhesion molecules and lectin-like oxidized LDL receptor-1 in human umbilical vein endothelial cells. Biosci Biotechnol Biochem.

[6] Ahmed F, Islam MA, Rahman MM (2006). Antibacterial activity of *Leonurus sibiricus* aerial parts. Fitoterapia.

[7] Sitarek P, Skała E, Toma M, Wielanek M, Szemraj J, Nieborowska- Skorska M (2016). A preliminary study of apoptosis induction in glioma cells via alteration of the Bax/Bcl-2-p53 axis by transformed and non-transformed root extracts of *Leonurus sibiricus* L. Tumour Biol.

[8] Sitarek P, Skała E, Toma M, Wielanek M, Szemraj J, Skorski T, Białas AJ, Sakowicz T, Kowalczyk T, Radek M, Wysokińska H, Śliwiński T (2016). Transformed root extract of *Leonurus sibiricus* induces apoptosis through intrinsic and extrinsic pathways in various grades of human glioma cells. Pathol Oncol Res.

[9] Sitarek P, Rijo P, Garcia C, Skała E, Kalemba D, Białas AJ, Szemraj J, Pytel D, Toma M, Wysokińska H, Śliwiński T (2017). Chemical composition antibacterial, anti-inflammatory, antioxidant and antiproliferative properties of essential oils from hairy and normal roots of *Leonurus sibiricus* L. Oxid Med Cell Longev.

[10] Sitarek P, Kowalczyk T, Rijo P, Białas AJ, Wielanek M, Wysokińska H, Garcia C, Toma M, Śliwiński T (2017). Skała E.. Over-expression of AtPAP1 transcriptional factor enhances phenolic acids production in transgenic roots of *Leonurus sibiricus* L. and their biological activities. Mol Biotechnol.

[11] Bouchard VJ, Rouleau M, Poirier GG (2003). PARP-1 a determinant of cell survival in response to DNA damage. Exp Hematol.

[12] Tanaka T, Kurose A, Huang X, Dai W, Darzynkiewicz Z (2006). ATM activation and histone H2AX phosphorylation as indicators of DNA damage by DNA topoisomerase I inhibitor topotecan and during apoptosis. Cell Prolif.

[13] Skała E, Kicel A, Olszewska MA, Kiss AK, Wysokińska H (2015). Establishment of hairy root cultures of *Rhaponticum carthamoides* (Willd.) Iljin for the Production of Biomass and Caffeic Acid Derivatives. BioMed Res Int.

[14] Czyż M, Toma M, Gajos-Michniewicz A, Majchrzak K, Hoser G, Szemraj J, Nieborowska-Skorska M, Cheng P, Gritsyuk D, Levesque M, Dummer R, Sliwinski T, Skorski T (2016). PARP1 inhibitor olaparib (Lynparza) exerts synthetic lethal effect against ligase 4-deficient melanomas. Oncotarget.

[15] Elmore S (2007). Apoptosis: a review of programmed cell death. Toxicol Pathol.

[16] Hassan M, Watari H, AbuAlmaaty A, Ohba Y, Sakuragi N (2014). Apoptosis and molecular targeting therapy in cancer. Biomed Res Int.

[17] De Saint-Hubert M, Bauwens M, Mottaghy FM (2014). Molecular imaging of apoptosis for early prediction of therapy efficiency. Curr Pharm Des.

[18] Ho JW, Cheung MW (2014). Combination of phytochemicals as adjuvants for cancer therapy. Recent Pat Anticancer Drug Discov.

[19] Nehybová T, Šmarda J, Beneš P (2014). Plant coumestans: recent advances and future perspectives in cancer therapy. Anticancer Agents Med Chem.

[20] Zhang Y, Yan YP, Wang ZZ (2010). The Arabidopsis PAP1 transcription factor plays an important role in the enrichment of phenolic acids in *Salvia miltiorrhiza*. J Agric Food Chem.

[21] De Carvalho MC, Barca FN, Aqnez-Lima LF, de Medeiros SR (2003). Evaluation of mutagenic activity in an extract of Pepper tree stem bark (*Schinus terebinthifolius* Raddi.). Environ Mol Mutag.

[22] Koňariková K, Ježovičová M, Keresteš J, Gbelcová H, Ďuračková Z, Žitňanová I (2015). Anticancer effect of black tea extract in human cancer cell lines. SpringerPlus.

[23] Aslanturk OS, Askin Çelik T (2013) Antioxidant, cytotoxic and apoptotic activities of extracts from medicinal plant *Euphorbia platyphyllos* L. J Med Plants Res 7(19), 1293–1304

[24] Burgos-Morón E, Calderón-Montaño JM, Orta ML, Pastor N, Pérez-Guerrero C, Austin C, Mateos S, López-Lázaro M (2012). The coffee constituent chlorogenic acid induces cellular DNA damage and formation of topoisomerase I– and II–DNA complexes in cells. J Agric Food Chem.

[25] Bürkle A, Brabeck C, Diefenbach J, Beneke S (2005). The emerging role of poly(ADP-ribose) polymerase-1 in longevity. Int J Biochem Cell Biol.

[26] Diaz-Hernandez JI, Moncada S, Bolaños JP, Almeida A (2007). Poly(ADP-ribose) polymerase-1 protects neurons against apoptosis induced by oxidative stress. Cell Death Differ.

[27] Mounira K, Nouha N, Imen M, Kamel G, Leila CG (2014) *Limoniastrum guyonianum* extracts induce apoptosis via DNA damage, PARP cleavage and UHRF1 down-regulation in human glioma U373 cells. J Nat Prod 7:79–86

[28] Robles-Escajeda E, Lerma D, Nyakeriga AM, Ross JA, Kirken RA (2013). Searching in mother nature for anti-cancer activity: anti- proliferative and pro-apoptotic effect elicited by green barley on leukemia/lymphoma cells. PLoS ONE.

[29] Lin H, Guo X, Zhang S, Dial SL, Guo L, Manjanatha MG, Moore MM, Mei N (2014). Mechanistic evaluation of *Ginkgo biloba* leaf extract-induced genotoxicity in L5178Y cells. Toxicol Sci.

[30] Tien AL, Senbanerjee S, Kulkarni A, Mudbghary R, Goudreau B, Ganesan S, Sadler KC, Ukomadu CH (2011). UHRF1 depletion causes a G2/M arrest, activation of DNA damage response and apoptosis. Biochem J.

[31] Alhosin M, Omran Z, Zamzami MA, Al-Malki AL, Choudhry H, Mousli M, Bronner CH (2016). Signalling pathways in UHRF1-dependent regulation of tumor suppressor genes in cancer. J Exp Clin Cancer Res.

[32] Arita K, Ariyoshi M, Tochio H, Nakamura Y, Shirakawa M (2008). Recognition of hemimethylated DNA by the SRA protein UHRF1 by a base-flipping mechanism. Nature.

[33] Muto M, Kanari Y, Kubo E, Takabe T, Kurihara T, Fujimori A, Tatsumi K (2002). Targeted disruption of Np95 gene renders murine embryonic stem cells hypersensitive to DNA damaging agents and DNA replication blocks. J Biol Chem.

[34] Mortusewicz O, Schermelleh L, Walter J, Cardoso MC, Leonhardt H (2005). Recruitment of DNA methyltransferase I to DNA repair sites. Proc Natl Acad Sci USA.

[35] Krifa M, Alhosin M, Muller CH-D, Gies J-P, Chekir-Ghedira L, Ghedira K, Mély Y, Bronner CH, Mousli M (2013). Limoniastrum guyonianum aqueous gall extract induces apoptosis in human cervical cancer cells involving p^16INK4A^ re-expression related to UHRF1 and DNMT1 down-regulation. J Exp Clin Cancer Res.

[36] Achour M, Mousli M, Alhosin M (2013). Epigallocatechin- 3-gallate up-regulates tumor suppressor gene expression via a reactive oxygen species-dependent down-regulation of UHRF1. Biochem Biophys Res Commun.

[37] Alhosin M, Leon-Gonzalez AJ, Dandache I (2015). Bilberry extract (Antho 50) selectively induces redox-sensitive caspase 3-related apoptosis in chronic lymphocytic leukemia cells by targeting the Bcl-2/Bad pathway. Sci Rep.

